# Accuracy of 3D-Scanned Full-Arch Dentition Versus 3D-Printed Models Generated Using Various 3D Printers Based on Linear Measurements

**DOI:** 10.7759/cureus.114449

**Published:** 2026-08-12

**Authors:** Soorabathula Sonika Mani Kiran, RSVM Raghu Ram, Inuganti Ranganayakulu, Buddha Sukumar, Neelapala Rohini, Ghanta Sunil

**Affiliations:** 1 Orthodontics and Dentofacial Orthopaedics, GSL Dental College & Hospital, Rajahmundry, IND

**Keywords:** 3d-printed dental models, 3d printers, intraoral scans, linear measurements, resins

## Abstract

Aim and objectives: To evaluate the accuracy of 3D-scanned full-arch dentition and 3D-printed models generated from various 3D printers and resin combinations using linear measurements.

Materials and methods: Intraoral scans of the mandibular arches of 30 patients were obtained using Primescan Dentsply Sirona (Dentsply Sirona Inc., USA/Germany), exported as Standard Tessellation Language (STL) files, and mesiodistal, intercanine, and intermolar width measurements were made using Maestro 3D Dental Studio software, version 6.0 (AGE Solutions S.r.l., Italy), as the reference standard. STL files were processed using Chitubox Basic software, version 2.3 (CBD-Tech (Shenzhen) Co., Ltd., China), and printed using stereolithography (SLA) 3D printers: Phrozen Sonic Mighty 4K (Phrozen Technology Co., Ltd., Taiwan) and Elegoo Saturn Ultra 4K (Shenzhen Elegoo Technology Co., Ltd., China), with Phrozen Aqua Gray 4K and Elegoo Standard photopolymer resins, yielding 120 models (30/group). Models were cleaned and cured using an Anycubic Cleaning and Curing Machine-Plus (Shenzhen Anycubic Technology Co., Ltd., China) and measured with Vernier calipers (Mitutoyo Corporation, Japan). Intra-examiner reliability was assessed after one week.

Results: Data were analyzed using one-way analysis of variance (ANOVA) and a post hoc Tukey test. The intraclass correlation coefficient (ICC) confirmed intra-examiner reliability (ICC > 0.98). No significant differences (p > 0.05) were observed between printed models and intraoral scans. The Elegoo printer with Phrozen resin showed the highest accuracy, while the Phrozen printer with Elegoo resin showed some deviation; all measurements were within clinically acceptable limits.

Conclusion: 3D-printed models showed comparable linear measurement accuracy to intraoral scans, with consistent reliability and clinically acceptable variations across printer-resin combinations.

## Introduction

Orthodontics has undergone significant transformation with the integration of digital technologies, especially 3D intraoral scanning and 3D printing. While conventional techniques have been fundamental in orthodontics, they present inherent challenges, including susceptibility to dimensional inaccuracies, fragility, and significant storage needs. Digital technologies address these limitations by offering high accuracy, faster workflows, and an improved patient experience. Intraoral scanners can produce highly detailed digital impressions that replicate dental anatomy with precision, providing a foundation for the fabrication of physical models using 3D printers [1,2].

Beyond their diagnostic capabilities, digital impressions serve as templates for creating physical models via 3D printing. This integration enables clinicians to transition seamlessly from diagnosis to appliance fabrication, significantly improving clinical outcomes and patient satisfaction [3,4]. This approach minimizes labor-intensive processes, reduces waste, and enhances treatment efficiency [5].

Dimensional accuracy, particularly in linear measurements such as mesiodistal widths, intercanine distances, and intermolar distances, is crucial for orthodontic treatment planning and appliance fabrication [6,7]. Even minor discrepancies in these measurements could lead to clinical errors, emphasizing the need for rigorous validation of 3D printing technologies [2,8]. Understanding these compatibilities is essential for selecting the optimal combination of technologies for specific clinical needs [9,10]. The ability of digital workflows to enhance diagnostic precision and streamline treatment planning is well established [11,12].

Factors such as layer thickness, curing time, and resin composition significantly influence the dimensional stability of printed models. Additionally, factors such as ambient temperature and resin viscosity can influence the precision of printed models, highlighting the importance of maintaining controlled environmental conditions during the printing process [13,14]. Addressing these aspects enables greater consistency in model production and minimizes errors in orthodontic diagnostics and appliance fabrication [15]. As orthodontics continues to integrate digital technologies, developing these protocols is crucial for building clinician confidence and promoting broader adoption [16,17].

The integration of 3D scanning and printing technologies marks a paradigm shift in orthodontics, offering unprecedented opportunities for innovation in diagnosis and treatment. Their ability to accurately replicate dental anatomy ensures the creation of appliances with superior fit and functionality [18]. This rapid workflow is particularly valuable in urgent clinical scenarios, such as addressing malocclusions or temporomandibular joint disorders that require immediate intervention [19].

While these technologies promise significant improvements in precision and efficiency, their successful implementation depends on rigorous validation of their accuracy and clinical relevance [20]. The purpose of this study is to evaluate and compare the accuracy of linear measurements, which are essential for diagnostic precision, treatment planning, and monitoring outcomes in orthodontics, obtained from 3D-scanned full-arch dentition models and 3D-printed models generated by various 3D printers and resin combinations.

## Materials and methods

This methodological validation study with a repeated-measures design was conducted in the Department of Orthodontics, GSL Dental College & Hospital, Rajahmundry, Andhra Pradesh, India. The study was approved by the GSL Dental College & Hospital Institutional Ethics Committee for Human Research on 08 April 2023 (Approval No. GSLDC/IEC/2023/19) and was prospectively registered with the Clinical Trials Registry-India (CTRI) under the Indian Council of Medical Research-National Institute for Research in Digital Health and Data Science (CTRI No. CTRI/2025/05/087486) before enrollment of the first participant. Participant recruitment was carried out between 10 June 2025 and 30 July 2025. A consecutive nonprobability sampling technique was employed to recruit eligible participants who attended the Department of Orthodontics seeking orthodontic treatment. The sample size was estimated a priori using G*Power software (version 3.1.9.7, Heinrich-Heine-University Düsseldorf, Germany). Assuming a large effect size (Cohen's f=0.40), a significance level (α) of 0.05, and a statistical power (1−β) of 80%, a minimum sample size of 30 participants was considered adequate for the study. Accordingly, 30 eligible participants who fulfilled the inclusion criteria were enrolled consecutively. All participants were informed about the purpose and procedures of the study, and written informed consent was obtained prior to enrollment.

The inclusion criteria were (1) no history of orthodontic treatment; (2) all fully erupted permanent teeth in the mandibular arch, excluding the third molars.

The exclusion criteria were (1) missing teeth in either arch; (2) morphological alterations of teeth that could affect the accuracy of the measurements; (3) decayed teeth, root canal-treated teeth, restorations, prostheses, or fractured teeth.

Patients were assessed using diagnostic instruments, including a mouth mirror and a straight probe, in accordance with the defined inclusion criteria. Individuals who met the inclusion criteria were informed about the study and the use of their intraoral scans.

3D-scanned full-arch dentition

**Figure 1:**
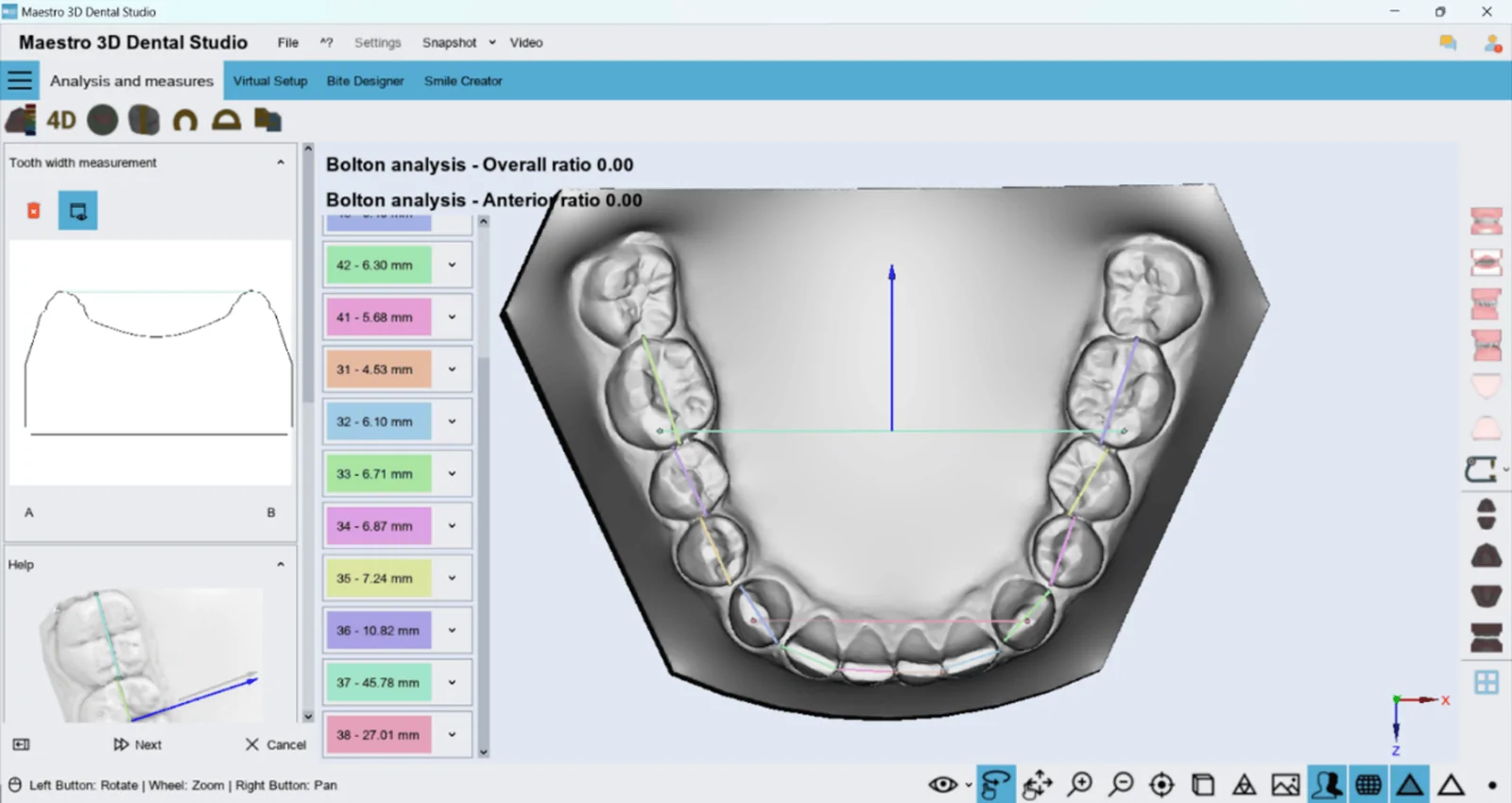
Measurements obtained using Maestro 3D software. Digital analysis performed with Maestro 3D Dental Studio software, version 6.0 (AGE Solutions S.r.l., Italy).

Intraoral scans of the mandibular arches of these 30 patients were captured using Primescan Dentsply Sirona (Dentsply Sirona Inc., USA/Germany) as part of their routine records at the time of their appointment and were exported as Standard Tessellation Language (STL) files. To maintain peak performance and ensure consistent precision, the scanner was calibrated weekly using a specialized calibration object. The data were analyzed, and linear measurements were made using Maestro 3D software, version 6.0 (AGE Solutions S.r.l., Italy), as shown in Figure 1, and these measurements were used as the reference for Group 1 (intraoral scan). Each participant contributed a single intraoral scan, which served as the reference dataset. The corresponding STL file from each participant was subsequently used to fabricate four different 3D-printed models using different printer-resin combinations. Thus, repeated measurements were obtained from the same reference dataset to compare the dimensional accuracy of the different printer-resin combinations.

3D-printed models

The STL files derived from the 3D scans of full-arch dentition were imported into Chitubox Basic, version 2.3 (CBD-Tech (Shenzhen) Co., Ltd., China), specialized slicing software designed for resin 3D printing. The digital models were precisely oriented on a flat plane on the virtual build platform to ensure structural integrity during printing and minimize the need for excessive support in critical anatomical areas, such as interproximal spaces and lingual undercuts.

In this study, two resin-based stereolithography (SLA) 3D printers, Elegoo Saturn Ultra 4K (Shenzhen Elegoo Technology Co., Ltd., China) and Phrozen Sonic Mighty 4K (Phrozen Technology Co., Ltd., Taiwan), were evaluated based on their machine configurations, print settings, and support parameters. Support structures were uniformly configured for both printers. The top support included a cone-shaped connection with a 0.30 mm tip diameter and 2 mm connection length. Middle supports featured a 0.80 mm cylindrical shape angled at 70°, with small cone-shaped pillars. Bottom supports had a skate-shaped platform touch with a 10 mm diameter and 0.80 mm thickness, with no raft structure but with the default raft values retained.

The sliced STL files from each participant were printed using the Phrozen Sonic Mighty 4K and Elegoo Saturn Ultra 4K printers with Phrozen Aqua Gray 4K and Elegoo Standard resins, producing a total of 120 printed models distributed equally among four experimental groups (30 models per group): Group 2 (Elegoo printer with Phrozen resin), Group 3 (Elegoo printer with Elegoo resin), Group 4 (Phrozen printer with Elegoo resin), and Group 5 (Phrozen printer with Phrozen resin). Accordingly, each participant contributed one model to each experimental group, enabling repeated measurements across all five study groups.

**Figure 2:**
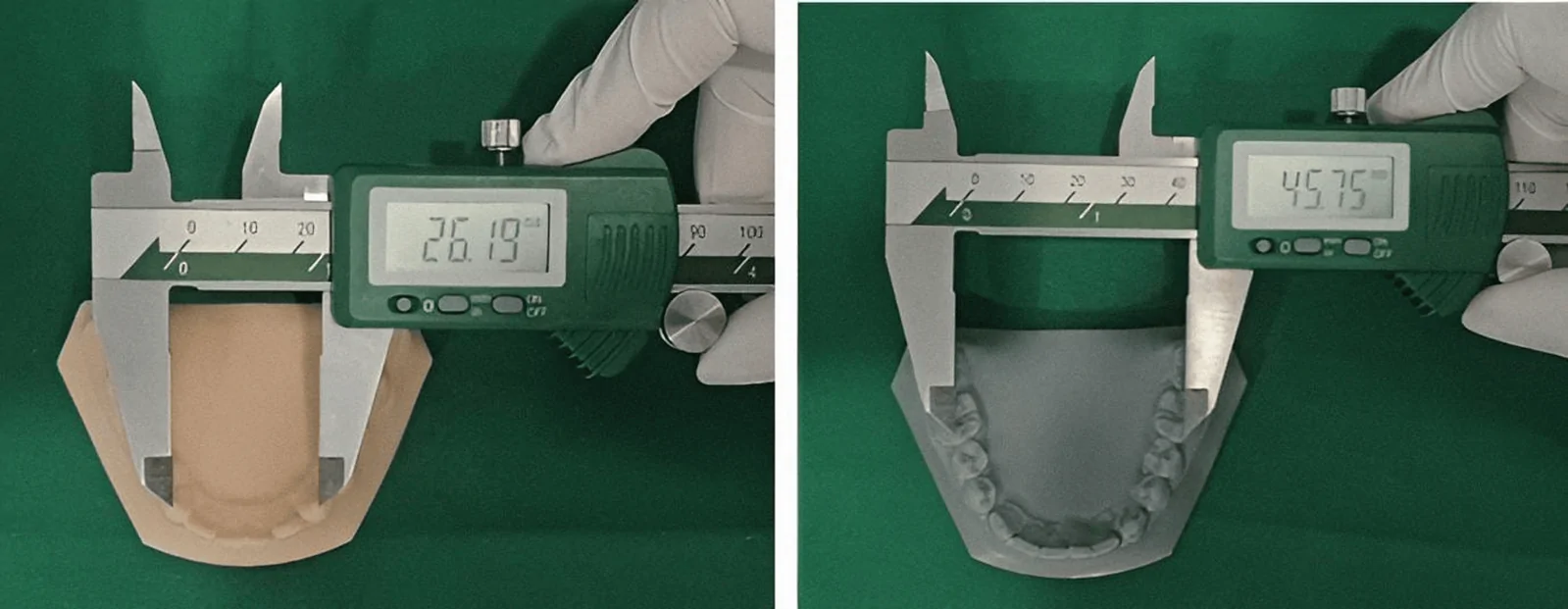
Measurements obtained from 3D-printed models.

In both of the above methods, mesiodistal tooth widths were recorded for the mandibular central incisors, lateral incisors, canines, premolars, and first molars at each tooth’s maximum contact-point width, with measurements taken parallel to the occlusal plane and perpendicular to the long axis to ensure consistency. Transverse mandibular arch width was assessed using intercanine width (ICW), measured between the cusp tips of the right and left canines, and intermolar width (IMW), measured between the mesiobuccal cusp tips of the right and left first molars, providing reliable indicators of the arch’s transverse dimension (Figure 2).

To minimize observer fatigue, no more than five pairs of models were measured during a single session. All measurements were performed in the same room under standardized artificial lighting conditions to ensure consistency. The STL files and corresponding 3D-printed models were assigned random identification codes, and the measurement order was randomized before analysis. The investigator performing the measurements was blinded to group allocation throughout the measurement procedure. By using identical measurement protocols for both tooth width and arch width assessments, the study maintained a high level of precision and repeatability.

Null hypothesis

The null hypothesis of this study was that there would be no statistically significant differences in the mesiodistal tooth width and transverse mandibular arch width measurements obtained from the reference intraoral scans (Group 1) and those obtained from the 3D-printed models fabricated using the four different printer-resin combinations (Groups 2-5).

Intra-examiner reliability was evaluated one week later by the same investigator. The final data were statistically analyzed using International Business Machines Corporation (IBM) Statistical Package for the Social Sciences (SPSS) software, version 26 (IBM SPSS, IBM Corp., Armonk, NY, USA). Because repeated measurements were obtained from the same participants across all study groups, comparisons were performed using repeated-measures analysis of variance (ANOVA). Post hoc pairwise comparisons were performed using Tukey's test. Intra-examiner reliability was assessed using the intraclass correlation coefficient (ICC). Statistical significance was set at P<0.05.

## Results

The comparison of mean values and their respective standard deviations across measurements is presented in Table 1. Across all measurements, the differences in means between the intraoral scan group and 3D-printed models were minimal. The slight variations in measurements among groups did not reach statistical significance, as indicated by high p-values (all >0.8). This suggests that models produced using different printers and resins closely replicate the accuracy of intraoral scans.

**Table 1:** Comparison of means of mesiodistal widths of various teeth, ICW, and IMW in the lower arch among study groups with sample size (N=30) for each group. One-way ANOVA; p≤0.05 was considered statistically significant. Group 1, intraoral scan; Group 2, Elegoo printer with Phrozen resin; Group 3, Elegoo printer with Elegoo resin; Group 4, Phrozen printer with Elegoo resin; Group 5, Phrozen printer with Phrozen resin. ICW, intercanine width; IMW, intermolar width; ANOVA, analysis of variance

Measurement	Group 1		Group 2		Group 3		Group 4		Group 5		F-value	df	Effect size	p-value
	Mean	SD	Mean	SD	Mean	SD	Mean	SD	Mean	SD				
31	5.16	0.55474	5.122	0.55405	5.112	0.55962	5.08	0.57316	5.112	0.55962	0.079	F (4,145)	0.002	0.989
32	5.936	0.5078	5.886	0.5022	5.876	0.493	5.866	0.4898	5.876	0.493	0.093	F (4,145)	0.003	0.984
33	7.138	0.89392	7.094	0.88314	7.086	0.88782	7.026	0.92406	7.058	0.90781	0.065	F (4,145)	0.002	0.992
34	7.17	0.73468	7.136	0.72563	7.122	0.72943	6.962	0.70321	7.06	0.73231	0.383	F (4,145)	0.01	0.821
35	7.248	0.57724	7.212	0.58351	7.198	0.58464	7.12	0.6324	7.17	0.59831	0.195	F (4,145)	0.005	0.941
36	10.81	0.55152	10.846	0.69985	10.832	0.70238	10.65	0.70905	10.832	0.70238	0.437	F (4,145)	0.012	0.782
41	5.464	0.51784	5.418	0.54781	5.39	0.51606	5.306	0.48581	5.39	0.51606	0.372	F (4,145)	0.01	0.829
42	6.04	0.4717	6.008	0.4595	5.998	0.4534	5.984	0.4515	5.998	5.998	0.063	F (4,145)	0.002	0.993
43	6.786	0.4717	6.726	0.4595	6.724	0.4534	6.676	0.4515	6.724	0.4534	0.096	F (4,145)	0.003	0.984
44	7.356	0.84176	7.316	0.85631	7.302	0.85381	7.21	0.84755	7.274	0.85246	0.123	F (4,145)	0.003	0.974
45	7.138	0.44238	7.074	0.43971	7.062	0.43588	7.028	0.47075	7.056	0.4383	0.251	F (4,145)	0.007	0.909
46	10.846	0.56719	10.82	0.563	10.808	0.55699	10.728	0.60012	10.78	0.56866	0.186	F (4,145)	0.005	0.945
ICW	27.108	1.23841	27.06	1.23675	27.052	1.23864	27.018	1.2368	27.042	1.23144	0.021	F (4,145)	0.001	0.999
IMW	44.21	1.75812	44.176	1.73779	44.166	1.73627	43.97	1.72743	44.078	1.72257	0.094	F (4,145)	0.003	0.984

Tukey's post hoc analysis in Table 2 further verified that there were no significant differences between the groups. The mean differences between the groups for all dependent variables were relatively small, and the significance values for all comparisons remained >0.05, indicating no statistically significant pairwise differences among groups. Although all values were statistically nonsignificant, some groups were closer to Group 1, while others showed more noticeable variations. Group 2 (Elegoo printer with Phrozen resin) consistently exhibited the smallest mean differences compared with Group 1 for most measurements. Group 4 (Phrozen printer with Elegoo resin) showed the greatest variations from Group 1 in several measurements, particularly measurements 34 and 36.

**Table 2:** Multiple pairwise comparisons of mean differences in mesiodistal widths of various teeth, ICW, and IMW in the mandibular arch among study groups. Tukey’s post hoc test; p≤0.05 was considered statistically significant. Group 1, intraoral scan; Group 2, Elegoo printer with Phrozen resin; Group 3, Elegoo printer with Elegoo resin; Group 4, Phrozen printer with Elegoo resin; Group 5, Phrozen printer with Phrozen resin; ICW, intercanine width; IMW, intermolar width

Measurement	Reference group (Group 1)								Standard error
	Comparison groups								
	Group 2		Group 3		Group 4		Group 5		
	Mean difference (I-J)	p-value	Mean difference (I-J)	p-value	Mean difference (I-J)	p-value	Mean difference (I-J)	p-value	
31	0.038	0.793	0.048	0.741	0.08	0.581	0.048	0.741	0.14466
32	0.05	0.698	0.06	0.641	0.07	0.586	0.06	0.641	0.1284
33	0.044	0.85	0.052	0.823	0.112	0.63	0.08	0.731	0.23224
34	0.034	0.856	0.048	0.798	0.208	0.268	0.11	0.558	0.18723
35	0.036	0.815	0.05	0.746	0.128	0.407	0.078	0.613	0.15377
36	0.036	0.837	0.022	0.9	0.16	0.361	0.022	0.9	0.17449
41	0.046	0.731	0.074	0.58	0.158	0.239	0.074	0.58	0.13351
42	0.032	0.787	0.042	0.723	0.056	0.637	0.042	0.723	0.1182
43	0.06	0.737	0.062	0.728	0.11	0.538	0.062	0.728	0.1782
44	0.04	0.856	0.054	0.806	0.146	0.507	0.082	0.709	0.21957
45	0.064	0.579	0.076	0.51	0.11	0.341	0.082	0.477	0.11505
46	0.026	0.86	0.038	0.797	0.118	0.425	0.066	0.655	0.14753
ICW	0.048	0.881	0.056	0.861	0.09	0.778	0.066	0.837	0.31924
IMW	0.034	0.94	0.044	0.922	0.24	0.593	0.132	0.769	0.44836

Intra-examiner reliability in Table 3 was assessed using an ICC with a two-way mixed-effects model, single measures, and absolute agreement (ICC 3,1), which showed excellent agreement across all measurements, with ICC values ranging from 0.980 to 0.994 and mean ICCs consistently >0.990. The P-values were <0.001 for all variables, indicating statistically significant reliability. The consistently high ICC values (>0.98) suggest excellent intra-examiner reliability, confirming that measurements were reproducible and consistent across different groups. This further supports the conclusion that intraoral scans and 3D-printed models maintain comparable levels of accuracy in orthodontic applications.

**Table 3:** Intra-examiner reliability assessed using the ICC. ICC (model: two-way mixed-effects, single measures, absolute agreement; ICC (3,1)). ***p<0.001 was considered statistically significant. Group 1, intraoral scan; Group 2, Elegoo printer with Phrozen resin; Group 3, Elegoo printer with Elegoo resin; Group 4, Phrozen printer with Elegoo resin; Group 5, Phrozen printer with Phrozen resin; ICC, intraclass correlation coefficient; ICW, intercanine width; IMW, intermolar width

Measurement	Group 1 ICC	Group 2 ICC	Group 3 ICC	Group 4 ICC	Group 5 ICC	Mean ICC	p-value
31	0.991	0.993	0.994	0.99	0.993	0.992	<0.001***
32	0.99	0.992	0.993	0.99	0.992	0.991	<0.001***
33	0.988	0.991	0.993	0.989	0.991	0.99	<0.001***
34	0.992	0.993	0.995	0.991	0.994	0.993	<0.001***
35	0.989	0.993	0.994	0.99	0.993	0.992	<0.001***
36	0.991	0.994	0.995	0.993	0.994	0.993	<0.001***
41	0.988	0.99	0.992	0.989	0.991	0.99	<0.001***
42	0.99	0.992	0.993	0.99	0.993	0.992	<0.001***
43	0.991	0.993	0.994	0.992	0.994	0.993	<0.001***
44	0.99	0.992	0.993	0.991	0.993	0.992	<0.001***
45	0.989	0.991	0.993	0.99	0.992	0.991	<0.001***
46	0.991	0.993	0.994	0.991	0.994	0.993	<0.001***
ICW	0.992	0.994	0.995	0.993	0.994	0.994	<0.001***
IMW	0.99	0.993	0.994	0.992	0.993	0.992	<0.001***

## Discussion

The integration of intraoral scanners and 3D printing technologies into orthodontics represents a paradigm shift in clinical workflows. This study addresses a critical gap in the literature by focusing on the accuracy of linear measurements obtained from 3D-printed models, evaluated using digital Vernier calipers and Maestro 3D software.

Precise linear measurements are critical in orthodontics, serving as the basis for diagnostics and treatment planning. Key measurements, such as tooth width, intercanine distance, and IMW, play a crucial role in evaluating the shape of the dental arch, diagnosing malocclusions, and designing orthodontic devices. Serrano-Velasco et al. supported this by validating the use of intraoral scanners and printed models for capturing intercanine and intermolar measurements in pediatric patients [21].

In this study, the measurements obtained digitally using Maestro 3D software were validated against physical measurements conducted with digital Vernier calipers to cross-check whether the printed models were generated accurately. Tsolakis et al. compared direct light processing (DLP) and liquid crystal display (LCD) printing technologies and observed that, while DLP printers generally exhibited slightly better dimensional accuracy, both technologies produced models that were within clinically acceptable limits for orthodontic use [22]. Tsolakis et al. showed that cost-effective LCD printers can also achieve acceptable dimensional trueness and reproducibility in dental model printing [23]. Igai et al. supported the influence of printer type on model trueness, with scanner choice having less impact [24].

Digital Vernier calipers were employed to physically measure mesiodistal widths, intercanine distances, and intermolar distances on 3D-printed models. The research showed that there were no notable discrepancies between the physical measurements and those obtained using the Maestro 3D software. This consistency supports the reliability of SLA printing technology for orthodontic applications. Park et al. further revealed that 3D-printed casts showed slightly greater volumetric changes than conventional casts, although they remained within acceptable clinical limits [25]. Suryajaya et al. further confirmed the effectiveness of 3D-printed models for conducting Bolton analysis and evaluating linear dental measurements [26]. Printing parameters such as layer thickness, build orientation, and curing protocols significantly influence the dimensional stability of 3D-printed models. Loflin et al. demonstrated that print layer height significantly affects the dimensional fidelity of printed models, with lower heights yielding more precise results [27].

The choice of resin plays a pivotal role in determining the dimensional accuracy of 3D-printed models. Phrozen Aqua Gray 4K and Elegoo Standard Photopolymer Resin, used in this study, demonstrated minimal shrinkage and excellent curing characteristics [3]. The Phrozen Aqua Gray 4K resin consistently maintained linear measurement deviations under 0.15 mm, highlighting its reliability for precision-demanding orthodontic applications. Similarly, the Elegoo Standard Photopolymer Resin offered reliable accuracy, demonstrating that budget-friendly materials can meet clinical demands when paired with optimized printing technologies. These outcomes align with observations by Etemad-Shahidi et al., who stressed that the characteristics of printing materials play a vital role in achieving accuracy in orthodontic 3D printing [3].

Physical measurements using digital Vernier calipers served as a robust validation method in this study. Repeated measurements over a one-week interval demonstrated high intra-observer reliability, with deviations consistently remaining within the clinically acceptable threshold of 0.5 mm. Choi et al. assessed inter-operator variability in intermaxillary relationships and confirmed printed model validity, although reproducibility could be influenced by operator skill [28].

Finally, the consistency in results between digital and physical measurements reinforces the clinical reliability of digital workflows, as noted by Pellitteri et al. [8] and Sherman et al. [10]. This alignment minimizes the need for redundant validation steps, streamlining appliance production and improving overall workflow efficiency.

Limitations

This study focused on a small sample size and only mandibular arches, limiting generalizability to diverse anatomies. The evaluation was restricted to two SLA printers and two resins, excluding other technologies such as DLP and fused deposition modeling (FDM). Manufacturer-recommended settings were used without exploring custom optimizations. Additionally, the potential for minor human error during physical measurements remains, despite ensuring intra-observer reliability.

Further scope

Future research should investigate the long-term dimensional stability of 3D-printed models under various storage and environmental conditions. Evaluating the performance of newer resin formulations and high-resolution printer technologies, such as monochromatic LCD printers, could provide valuable insights into optimizing orthodontic workflows. Advancements in materials science and 3D printing technology are expected to improve the precision and scalability of digital workflows.

## Conclusions

3D-printed models exhibited no significant variation in linear measurement accuracy compared with 3D-scanned full-arch dentition across all printer-resin combinations.

All 3D-printed models exhibited similar dimensional accuracy, with the Elegoo printer with Phrozen resin combination showing the highest concordance with intraoral scans, while the Phrozen printer with Elegoo resin combination showed slightly higher yet clinically acceptable deviations.
